# Impact of Proton versus Photon (Chemo)radiation on Circulating Immune Cells in Head and Neck Cancer

**DOI:** 10.1158/2767-9764.CRC-26-0496

**Published:** 2026-09-08

**Authors:** Anneke L. Eerkens, Nienke van Rooij, Annechien Plat, Annegé Vledder, Koen Brummel, Sjoukje F. Oosting, Bert van der Vegt, Gyorgy B. Halmos, Johannes A. Langendijk, Marco de Bruyn, Hans W. Nijman, Tineke W.H. Meijer

**Affiliations:** 1 Department of Obstetrics and Gynaecology, University Medical Center Groningen, Groningen, the Netherlands.; 2 Department of Medical Oncology, University Medical Center Groningen, Groningen, the Netherlands.; 3 Department of Pathology and Medical Biology, University Medical Center Groningen, Groningen, the Netherlands.; 4 Department of Otorhinolaryngology – Head and Neck Surgery, University Medical Center Groningen, Groningen, the Netherlands.; 5 Department of Radiation Oncology, University Medical Center Groningen, Groningen, the Netherlands.

## Abstract

**Purpose:**

Proton therapy delivers more precise radiation (RT) with less toxicity than photons. However, its impact on circulating immune cells in head and neck squamous cell carcinoma (HNSCC) remains poorly understood. We compared the impact of proton versus photon RT on circulating immune cells in patients with locally advanced HNSCC undergoing definitive (chemo)radiation [(C)RT].

**Patients and Methods:**

Patients with stage III to IV HNSCC receiving definitive RT (70 Gy) with or without weekly cisplatin using proton or photon therapy were enrolled. Peripheral blood was collected at baseline, during treatment, and up to 1 year after treatment. White blood cell differentiation, lymphocyte subsets, and lymphocyte function were evaluated using high-dimensional flow cytometry and functional assays.

**Results:**

Twenty-seven patients were enrolled: Ten received CRT with protons, eight received RT alone with protons, and nine received RT alone with photons. Absolute lymphocyte counts and naïve CD4^+^ T cells declined in all groups, whereas NK cells increased and memory T-cell populations remained largely unaffected, with no additional effect of concurrent cisplatin. Lymphocyte suppression persisted for up to 6 months in the CRT proton group, 3 months in the RT proton group, and 12 months in the RT photon group. Greater low-dose body irradiation (2 Gy) was correlated with lower lymphocyte counts at 5 weeks and 6 months after treatment and was more common in photon-treated patients.

**Conclusions:**

Both proton and photon RT markedly suppress circulating lymphocytes, particularly naïve CD4^+^ T cells, for at least 3 months after treatment in locally advanced HNSCC.

**Significance:**

Proton RT is increasingly utilized for head and neck cancer. However, its impact on systemic immunity remains unclear. We demonstrated that both proton and photon RT caused prolonged suppression of circulating lymphocytes for at least 3 months. Naïve CD4^+^ T cells were preferentially depleted, whereas memory T-cell populations were largely spared. These findings highlight the importance of considering immune effects and minimizing off-target lymphocyte exposure when combining radiotherapy with immunotherapy.

## Introduction

Head and neck squamous cell carcinoma (HNSCC) is the seventh most commonly diagnosed cancer worldwide, with an estimated annual incidence of 890,000 cases (1). Standard-of-care treatment varies based on disease stage, anatomic site, and surgical accessibility (2). It typically involves primary surgery with or without postoperative (chemo)radiation [(C)RT] or primary (C)RT. CRT provides better tumor control compared with radiation (RT) alone in locally advanced disease but is associated with increased toxicity. However, the overall survival (OS) of locally advanced HNSCC remains poor, with a 5-year OS of 50% (2).

Immune checkpoint inhibitors (ICI) targeting the programmed cell death protein 1 (PD-1), programmed cell death ligand 1 (PD-L1), and cytotoxic T lymphocyte (CTL)–associated protein 4 (CTLA-4) pathways have transformed cancer therapy and are approved for recurrent and metastatic HNSCC. More recently, in the perioperative setting, ICIs have demonstrated improved event-free survival (3). However, trials combining ICIs with primary (chemo)radiotherapy (4–6) failed to show meaningful improvements in OS and progression-free survival.

One potential explanation for the limited efficacy of ICIs in combination with (chemo)radiotherapy is the high radiosensitivity of lymphocytes to photon RT. Even a low dose of 2 Gy can lead to the death of more than 50% of the irradiated lymphocytes (7). Additionally, photon RT can significantly impair T-cell function, reducing their ability to respond to antigens for up to 6 months after treatment (8, 9). Although chemotherapy may affect lymphocyte counts, studies directly comparing RT alone with RT plus concurrent cisplatin have shown that lymphocyte depletion is primarily driven by RT, with cisplatin neither increasing the extent nor altering the pattern of lymphocyte loss (10).

Recent advancements in HNSCC treatment include the use of proton therapy. Owing to its superior dose distribution and reduced RT exposure to adjacent normal tissues, proton therapy may help mitigate treatment-related lymphocyte depletion. Consistent with this, studies in patients with glioblastoma, esophageal cancer, lung cancer, and hepatocellular carcinoma have reported lower rates of lymphopenia after proton therapy compared with photon therapy (11–17).

Hence, understanding the differential immunomodulatory effects of proton and photon RT is essential for optimizing the synergy between radiotherapy and immunotherapy. In this study, we aim to compare the impact of proton versus photon RT on the composition and function of circulating immune cells in patients with HNSCC undergoing primary RT or CRT.

## Patients and Methods

### Study design

This single-institution investigator-initiated prospective observational pilot study was carried out in the Netherlands at the University Medical Center Groningen (UMCG). The study protocol and all amendments were approved by the independent ethics committee of the UMCG. The study was conducted in accordance with the protocol, the Declaration of Helsinki, and the International Conference on Harmonization Guidelines for Good Clinical Practice. All patients provided written informed consent. The trial is registered at ClinicalTrial.gov (NCT06016699).

### Patient population

Patients were eligible if they were 18 years or older; had pathologically and radiologically confirmed stage III to IV HNSCC of the oral cavity, oropharynx, hypopharynx, or larynx; and were intended to be treated with standard-of-care definitive RT with photons (volumetric-modulated arc therapy) or protons (intensity-modulated proton therapy; 70 Gy) with or without weekly cisplatin 40 mg/m^2^. In the Netherlands, patients qualify for proton therapy if they meet the criteria of the national indication protocol for proton therapy (18). According to our standard-of-care protocol, patients younger than 70 years could be considered eligible for CRT, whereas patients ≥70 years received RT alone. Key exclusion criteria were unilateral irradiation of the neck (due to the lower overall RT exposure compared with bilateral treatment), resection of the primary tumor, treatment involving a combination of proton and photon RT, treatment with immunosuppressive medication, recent systemic anticancer therapy, active autoimmune disease and other active cancer, immunodeficiency, and pregnancy.

### Blood sampling and processing

Circulating immune cells were collected at baseline (T1), during the course of (C)RT between the third and fourth week of treatment (T2), and at follow-up at 2, 5, 12 (i.e., 3 months), 26 (i.e., 6 months), and 52 (i.e., 1 year) weeks after (C)RT (T3–T7, respectively). At each collection time point, 4 mL of peripheral blood was drawn into Sysmex tubes (BD Vacutainer) for routine white blood cell (WBC) count and differential analysis, whereas 100 mL of peripheral blood was collected in lithium heparin tubes (BD Vacutainer) for the isolation of peripheral blood mononuclear cells (PBMC). PBMCs were isolated using Ficoll density gradient centrifugation (Ficoll-Paque PLUS, Cytiva). The isolated cells were cryopreserved in a solution containing 90% fetal calf serum (FCS; Bodinco BV) and 10% dimethyl sulfoxide (DMSO; Merck) at a concentration of 5 to 20 × 10^6^ cells per cryovial at −180°C until further experiments were conducted.

### Spectral flow cytometry

Fluorescence-activated cell sorting (FACS) analysis was done using a Cytek Aurora flow cytometer [Cytek Aurora Spectral Analyzer (RRID: SCR_019826)] with five flat-top lasers (UV, 350 nm; violet, 405 nm; blue, 488 nm; yellow–green, 561 nm; and red, 640 nm). For sample preparation, cryopreserved PBMCs were thawed in FCS, washed in phosphate-buffered saline (PBS), and resuspended at a density of 2 × 10^6^ cells per FACS tube. Then, cells were incubated with a live/dead marker (100 μL of 1:100 diluted in PBS per sample; Thermo Fisher Scientific, cat. #L23105, RRID: AB_3717566) for 20 minutes at room temperature in the dark. After washing the cells with PBS supplemented with 5% FCS, cells were blocked by incubation with Human TruStain FcX receptor (10 μL per sample; BioLegend, cat. #422302, RRID: AB_281898) and CellBlox blocking buffer (5 μL per sample; Thermo Fisher Scientific). Next, cells were first stained with TCRg/d-PerCP-eFluor710 (5 μL per sample; Thermo Fisher Scientific, cat. #46-9959-41, RRID: AB_2573925) for 10 minutes at room temperature in the dark, followed by incubation with CXCR5-BV750 (1.25 μL; BD Biosciences, cat. #747111, RRID: AB_2871862) for 10 minutes at room temperature in the dark. Thereafter, the antibody master mix, consisting of markers, for example, T cells, B cells, NK cells, and myeloid cells (see Supplementary Table S1 for the full list and antibody characteristics), was added to the samples. After incubation with the master mix for 30 minutes in the dark on ice, cells were washed twice with PBS supplemented with 2% FCS and kept on ice in the dark. Within 3 hours after preparation, samples were measured using the Cytek Aurora. For each patient, an unstained sample served as a negative control.

Data acquisition and quality control were done using the SpectroFlo software (version 3.0; RRID: SCR_025494). Data were analyzed using CITRUS on Cytobank software (version 10.2.4; RRID: SCR_014043). CITRUS first clustered cells based on marker expression across all PBMCs and calculated relative abundance for each cluster as the proportion of cells within the cluster relative to the total PBMC count (values between 0 and 1). For visualization, only clusters showing significant increases over time in response to treatment were displayed in the figures.

### Enzyme-linked immunosorbent spot assays

Memory T-cell responses during and after (C)RT were assessed by *ex vivo* enzyme-linked immunosorbent spot (ELISpot) assay. On day 1, cryopreserved PBMCs were thawed, resuspended at a concentration of 2 × 10^6^ cells/mL in a 50-mL tube in medium consisting of X-VIVO 15 (Lonza Bioscience) supplemented with 1% penicillin/streptomycin (Thermo Fisher Scientific) and 1% L-glutamine (2 mmol/L; Thermo Fisher Scientific), and rested overnight at 37°C and 5% CO_2_. Multiscreen 96-well ELISpot plates (Millipore) were coated with anti-human IFNγ coating antibody (5 μg/mL diluted in PBS; mAB-1-DK; Mabtech, cat. #3420-2APT, RRID: AB_2877719) and incubated overnight at 4°C. On day 2, ELISpot plates were washed with PBS and blocked with the medium for 2 hours at 37°C. Rested PBMCs were added to the precoated wells at a density of 300,000 cells/well in 100 μL of medium and tested for activity against viral peptide pools (1 μg/mL/peptide). The viral peptides used included the severe acute respiratory syndrome coronavirus 2 (SARS-CoV-2) peptide pool [PM-WCPV-S-2 (vial 1 + 2); JPT Peptide Technologies], comprising 315 peptides derived from the spike glycoprotein of SARS-CoV-2, and the CEF peptide pool (PM-CEF-S-4; JPT Peptide Technologies), consisting of 23 viral peptides derived from human cytomegalovirus, Epstein–Barr virus, and influenza A virus. DMSO served as a negative control and phytohemagglutinin (5 μg/mL; Sigma-Aldrich) as a nonspecific positive control. After incubation for 16 to 24 hours with peptides, the contents of the plates were discarded, and the plates were washed thoroughly with washing buffer, consisting of PBS supplemented with 0.1% Tween 20 (Sigma-Aldrich). Then, wells were incubated with biotinylated anti-human IFNγ antibody (0.3 μg/mL; mAB-7-B6-biotin; Mabtech, cat. #3420-2APT, RRID: AB_2877719) for 2 hours at room temperature in the dark and washed thoroughly with washing buffer. Following incubation of the wells with ExtrAvidin-Alkaline Phosphatase (ALP; 1 μg/mL in PBS; Sigma-Aldrich) for 1 hour at room temperature in the dark, plates were washed with running distilled water. Subsequently, the wells were incubated with the substrate 5-bromo-4-chloro-3-indolyl phosphate/nitroblue tetrazolium-ALP (Sigma-Aldrich) until staining was visually apparent (3–8 minutes). The wells were then thoroughly washed under running distilled water and allowed to dry overnight at 4°C in the dark. Spot enumeration was performed using an AID EliSpot reader (AID Autoimmun Diagnostika; RRID: SCR_025287).

### Statistical analysis

Statistical analysis was performed using GraphPad Prism (version 9; RRID: SCR_002798). Differences in baseline patient characteristics between groups were assessed using Kruskal–Wallis tests for numeric variables and *χ*^2^ tests for categorical variables (Table 1). Changes in the absolute number of circulating immune cell subsets over time within treatment groups were analyzed by comparing values during treatment and at follow-up relative to baseline levels using a mixed-effects model with Geisser–Greenhouse correction. Differences in the absolute number of circulating immune cell subsets between treatment groups were evaluated using a mixed-effects model with Geisser–Greenhouse correction, with the RT photon group designated as the reference group. Differences in RT volume between treatment groups were assessed using the Kruskal–Wallis test. Spearman correlation analysis was performed to evaluate the relationship between absolute lymphocyte counts and RT volume. Differences in memory T-cell responses between visits were analyzed using a mixed-effects model with Bonferroni correction, with visit 1 serving as the reference. A *P* value <0.05 was considered to be statistically significant.

**Table 1.** Baseline characteristics of the patients within the PRO-IMMUNO trial.

Characteristic	Treatment group			
	CRT proton (*n* = 10)	RT proton (*n* = 8)	RT photon (*n* = 9)	*P* value
Age at enrollment (years)	​	​	​	​
Median (range)	58 (35–69)	70 (58–77)	73 (63–88)	**<0.001** ^a^
Gender [*n* (%)]	​	​	​	​
Male	5 (50)	6 (75)	7 (78)	0.368
Female	5 (50)	2 (25)	2 (22)	​
PS [*n* (%)]	​	​	​	​
0	10 (100)	6 (75)	2 (22)	**<0.007** ^a^
1	0 (0)	2 (25)	5 (56)	​
2	0 (0)	0 (0)	2 (22)	​
Alcohol (units/week)	​	​	​	​
Median (range)	2 (0–11)	1.5 (0–21)	3 (0–35)	0.677
Smoking (pack-years)	​	​	​	​
Median (range)	2.5 (0–52)	30 (0–125)	25 (1–60)	0.094
p16 status [*n* (%)]	​	​	​	​
Positive	6 (60)	3 (38)	2 (22)	0.241
Negative	4 (40)	5 (62)	7 (78)	​
Tumor site [*n* (%)]	​	​	​	​
Oral cavity	2 (20)	2 (25)	1 (11)	**0.037** ^b^
Oropharynx	8 (80)	6 (75)	2 (22)	​
Hypopharynx	0 (0)	0 (0)	2 (22)	​
Larynx	0 (0)	0 (0)	4 (45)	​
T [*n* (%)]^c^	​	​	​	​
T1	2 (20)	0 (0)	1 (11)	0.267
T2	2 (20)	1 (12)	3 (33)	​
T3	0 (0)	3 (38)	3 (33)	​
T4	6 (60)	4 (50)	2 (22)	​
N [*n* (%)]^c^	​	​	​	​
N0	0 (0)	1 (12)	4 (45)	0.221
N1	3 (30)	2 (25)	1 (11)	​
N2	5 (50)	2 (25)	3 (33)	​
N3	2 (20)	3 (38)	1 (11)	​

Bold values indicate statistically significant differences (*P* < 0.05).

a Significant difference between CRT proton and RT photon groups, *P* < 0.05.

b Significant difference between CRT proton and RT proton groups and between RT proton and RT photon groups; *P* < 0.05.

c Tumor–node–metastasis staging system according to the eighth edition of the American Joint Committee on Cancer.

## Results

### Patient characteristics

**Figure 1.**
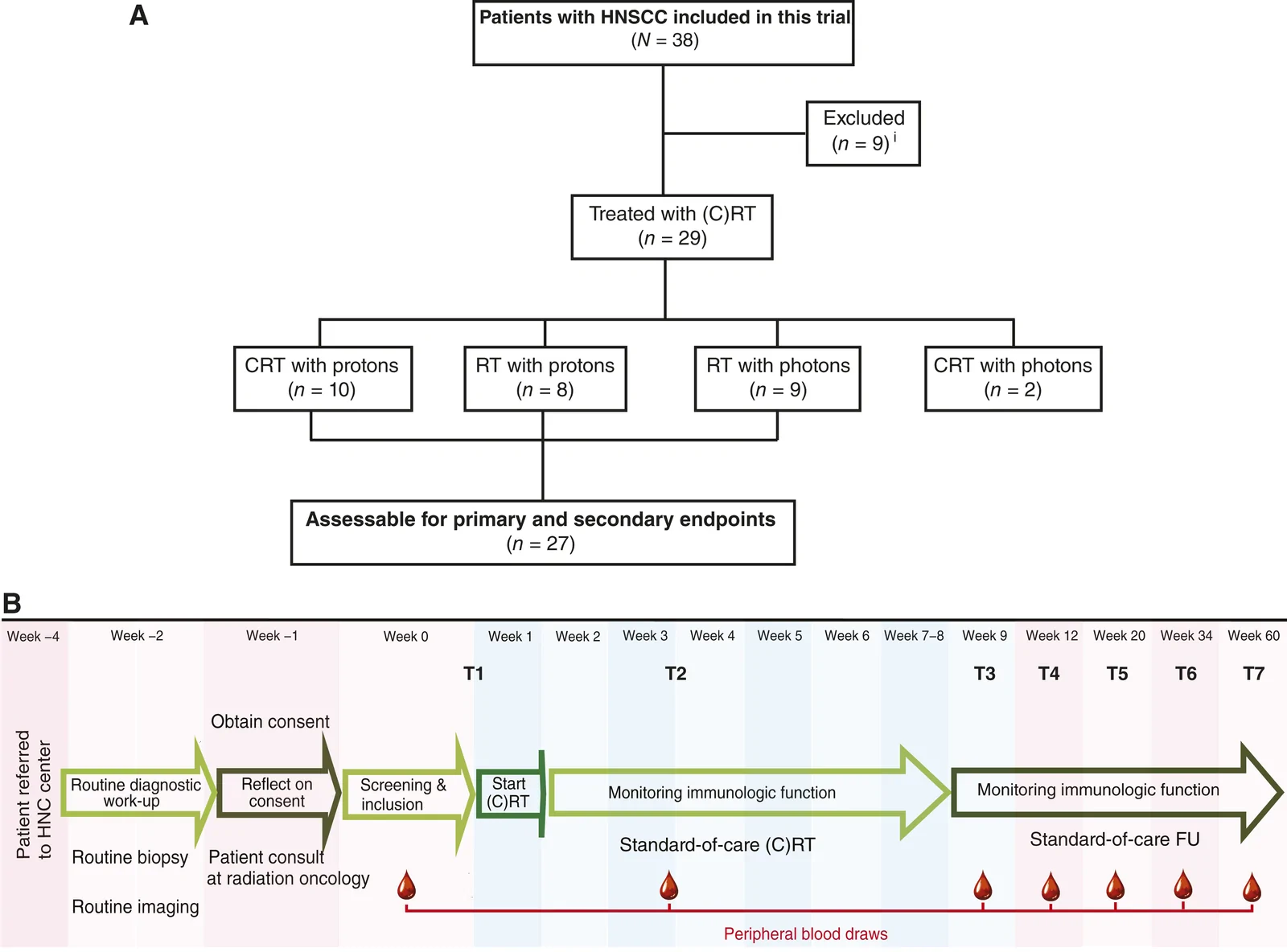
Consort diagram and study design. **A,** Consort diagram depicting the disposition of patients throughout the study and treatment groups. Thirty-eight patients were recruited to this study, of whom 27 were assessable for our analysis. (i) Reasons for exclusion from our analysis were withdrawal of consent, death during the trial, referral to an alternative medical facility, and being subjected to a combination of proton and photon RT regimen. **B,** Study design. Patients with pathologically and radiologically confirmed stage III–IV HNSCC of the oral cavity, oropharynx, hypopharynx, or larynx were treated with standard-of-care definitive RT with protons or photons (70 Gy) with or without weekly cisplatin. FU, follow-up; HNC, head and neck cancer.

A total of 38 patients with histologically proven HNSCC stages III to IV, who were to receive definitive CRT or RT either with protons or photons, were enrolled between September 2021 and November 2023 (Fig. 1A and B). Nine patients were excluded due to dropout or because they had received treatment involving a combination of proton and photon RT. Of the 29 remaining patients, 10 underwent CRT with protons, 8 received RT alone with protons, 2 received CRT with photons, and 9 received RT alone with photons. As only two patients received CRT with photons, these were excluded from subsequent analyses.

Table 1 outlines the baseline characteristics of the patients. The median age was 58 years in the CRT proton group, 70 years in the RT proton group, and 73 years in the RT photon group. Most patients were male (67%), had T3 to T4 tumors (67%), and exhibited image-confirmed metastasis to the lymph nodes (74%). Patients who received CRT were younger and had a better performance status (PS) than patients who received RT (either protons or photons) alone. Tumor–node–metastasis stage, median alcohol intake (units/week), and smoking habits (pack-years) were balanced between groups. The primary tumor site differed between the proton and photon groups. The oropharynx was the most common site in both proton therapy groups (80% in the CRT and 75% in the proton therapy alone group), whereas the larynx was the most common site in the photon therapy group (45%). Bold values indicatie statistically significant differences (*P *<0.05). The representativeness of the study participants is summarized in Supplementary Table S2.

### Effect of proton and photon radiotherapy on the absolute number of circulating immune cell subsets

**Figure 2.**
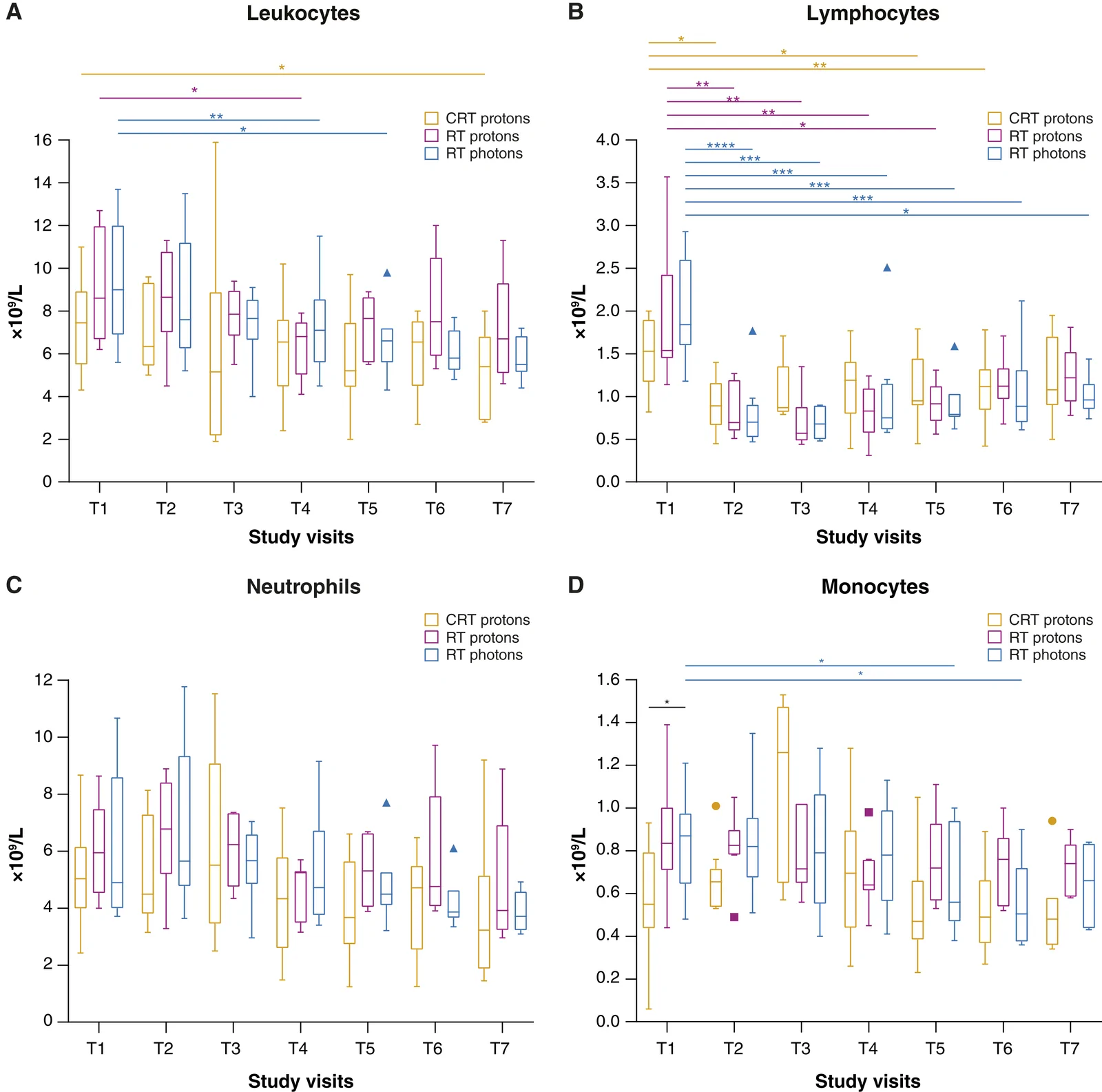
Absolute number of circulating immune cells at baseline, during, and after RT (up to 12 months after completion of treatment) using protons compared with photons. **A–D,** Tukey box plots showing the (**A**) leukocyte counts, (**B**) lymphocyte counts, (**C**) neutrophil counts, and (**D**) monocyte counts in patients treated with CRT with protons, RT with protons alone, and RT with photons alone at baseline, during treatment, and following treatment from 2 weeks up to 12 months after treatment. *, *P* ≤ 0.05; **, *P* ≤ 0.01; ***, *P* ≤ 0.001; ****, *P* ≤ 0.0001. T1, visit 1 (baseline); T2, visit 2 (during treatment); T3–7, visits 3–7 (follow-up visits at 2–52 weeks after completion of treatment, respectively). The symbols (triangle, square, and circle) in the figure represent outliers.

For all 27 patients, WBC counts were available from at least T1 (baseline) to T6 (6 months after treatment; Fig. 2). Although no significant differences were observed between treatment groups over time, each group showed a significant decrease in leukocyte and lymphocyte counts following treatment compared with baseline values (Fig. 2A and B). Notably, the number of lymphocytes remained below baseline for up to 12 months in the RT photon group, up to 6 months in the CRT proton group, and up to 3 months in the RT proton group (Fig. 2B). Neutrophil counts remained stable, with no differences detected either between or within the treatment groups over time (Fig. 2C). Monocyte counts were significantly lower at baseline in the CRT proton group compared with the RT photon group (Fig. 2D). A significant decrease in monocyte counts was only observed in the RT photon group, lasting for up to 6 months after treatment (Fig. 2D). Concurrent weekly cisplatin did not seem to further exacerbate the magnitude of lymphocyte depletion.

Taken together, these data indicate that both proton and photon radiotherapy exert long-lasting immunosuppressive effects, primarily on lymphocytes, with the duration of suppression longest following photon therapy.

### Effect of radiation volume and dose on circulating lymphocyte count

**Figure 3.**
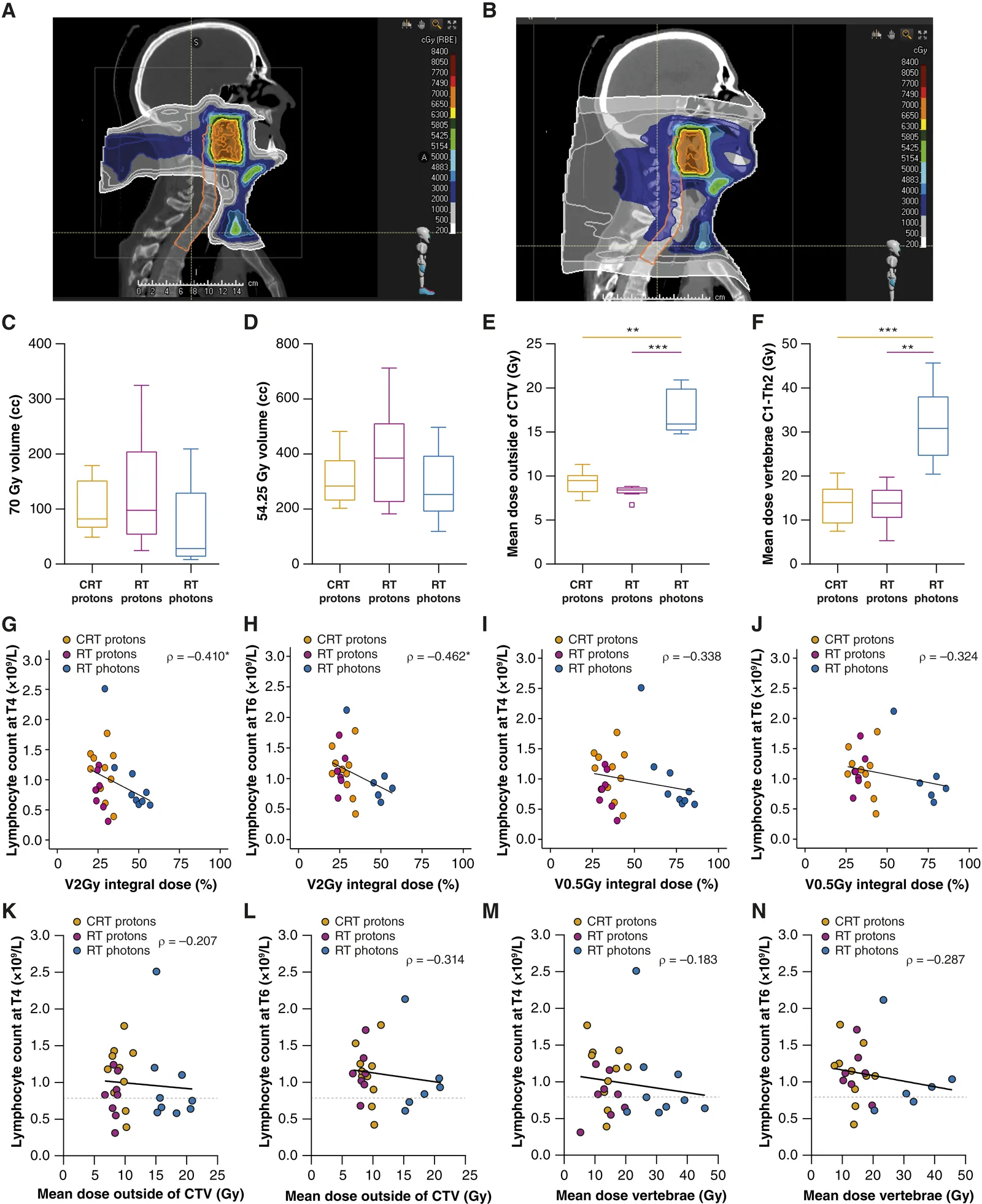
RT dose and body volume in relation to absolute lymphocyte counts. **A** and **B,** Representative RT therapy plan for a patient with HNSCC in the study treated with (**A**) proton therapy and (**B**) photon therapy. **C–F,** Tukey box plots showing the differences in (**C**) CTV receiving a boost dose (70 Gy), (**D**) CTV receiving an elective treatment dose (54.25 Gy), (**E**) dose administered to the area outside of the CTV spanning from the sphenoid sinus to the sternum, and (**F**) dose administered to the vertebral bodies of the cervical spine among patients subjected to CRT with protons, RT with protons alone, and RT with photons alone. **G** and **H,** Scatterplots showing the correlation between the percentage of body volume receiving 2 Gy of RT and absolute lymphocyte counts at (**G**) T4 and (**H**) T6. **I** and **J,** Scatterplots showing the correlation between the percentage of body volume receiving 0.5 Gy of RT and absolute lymphocyte counts at (**I**) T4 and (**J**) T6. **K** and **L,** Scatterplots representing the correlation between RT dose administered outside of the CTV and absolute lymphocyte counts at (**K**) T4 and (**L**) T6. **M** and **N,** Scatterplots representing the correlation between RT dose administered to the vertebral bodies of the cervical spine and absolute lymphocyte counts at (**M**) T4 and (**N**) T6. *, *P* ≤ 0.05; **, *P* ≤ 0.01; ***, *P* ≤ 0.001. ρ, Spearman correlation coefficient. The dotted line denotes the lower boundary for absolute lymphocyte counts. T4 and T6, visit 4 and 6, follow-up visits at 5 weeks and 6 months after completion of treatment, respectively. cGy, centigray; RBE, relative biological effectiveness.

RT volume and dose have been linked to a decrease in lymphocyte counts (19, 20). We therefore compared the RT volumes delivered to the clinical target volume (CTV) receiving 70 Gy (primary tumor and pathologic lymph nodes), the elective areas receiving 54 Gy, the mean RT dose delivered to areas outside the CTV, and the mean RT dose delivered to the vertebral bodies of the cervical spine across treatment groups (Fig. 3A and B). RT volume administered to the CTV and elective areas was not significantly different among treatment groups (Fig. 3C and D). However, proton therapy resulted in significantly lower mean RT doses administered to areas outside of the CTV (CRT protons, 9.20 Gy; RT protons, 8.23 Gy) compared with photon therapy (17.31 Gy). Similarly, mean doses to the vertebral bodies of the cervical spine were significantly lower with protons (CRT protons, 13.64 Gy; RT protons, 13.33 Gy) than with photons (31.34 Gy; Fig. 3E and F).

To evaluate the potential impact of low-dose exposure to larger body volumes on lymphocyte counts during and after treatment, we analyzed integral dose–volume parameters. These were expressed as the percentage of body volume from the vertex to the mid-thoracic level receiving 0.5 and 2 Gy, respectively (V0.5 Gy and V2 Gy integral dose; Fig. 3G–J; Supplementary Fig. S1A–S1H). V2 Gy showed a significant inverse correlation with lymphocyte counts at T4 and T6 (Fig. 3G and H), indicating that patients with a larger proportion of body volume exposed to 2 Gy tended to have lower lymphocyte counts at follow-up. These patients were predominantly treated with photon therapy, consistent with the larger low-dose irradiated volumes of photon-based techniques, as also reflected in the higher mean doses to the vertebral bodies and areas outside of the CTV (Fig. 3E and F). In contrast, no significant correlations were observed for V0.5 Gy. Likewise, the RT dose to areas outside of the CTV and to the vertebral bodies of the cervical spine did not show significant correlations with lymphocyte counts when analyzed separately (Fig. 3K–N; Supplementary Fig. S1I–S1P). These findings suggest that low-dose exposure across a larger body volume, as captured by V2 Gy, is more relevant for its association with lymphocyte dynamics than isolated organ-specific dose metrics.

### Effect of proton and photon radiotherapy on circulating lymphocyte subsets

**Figure 4.**
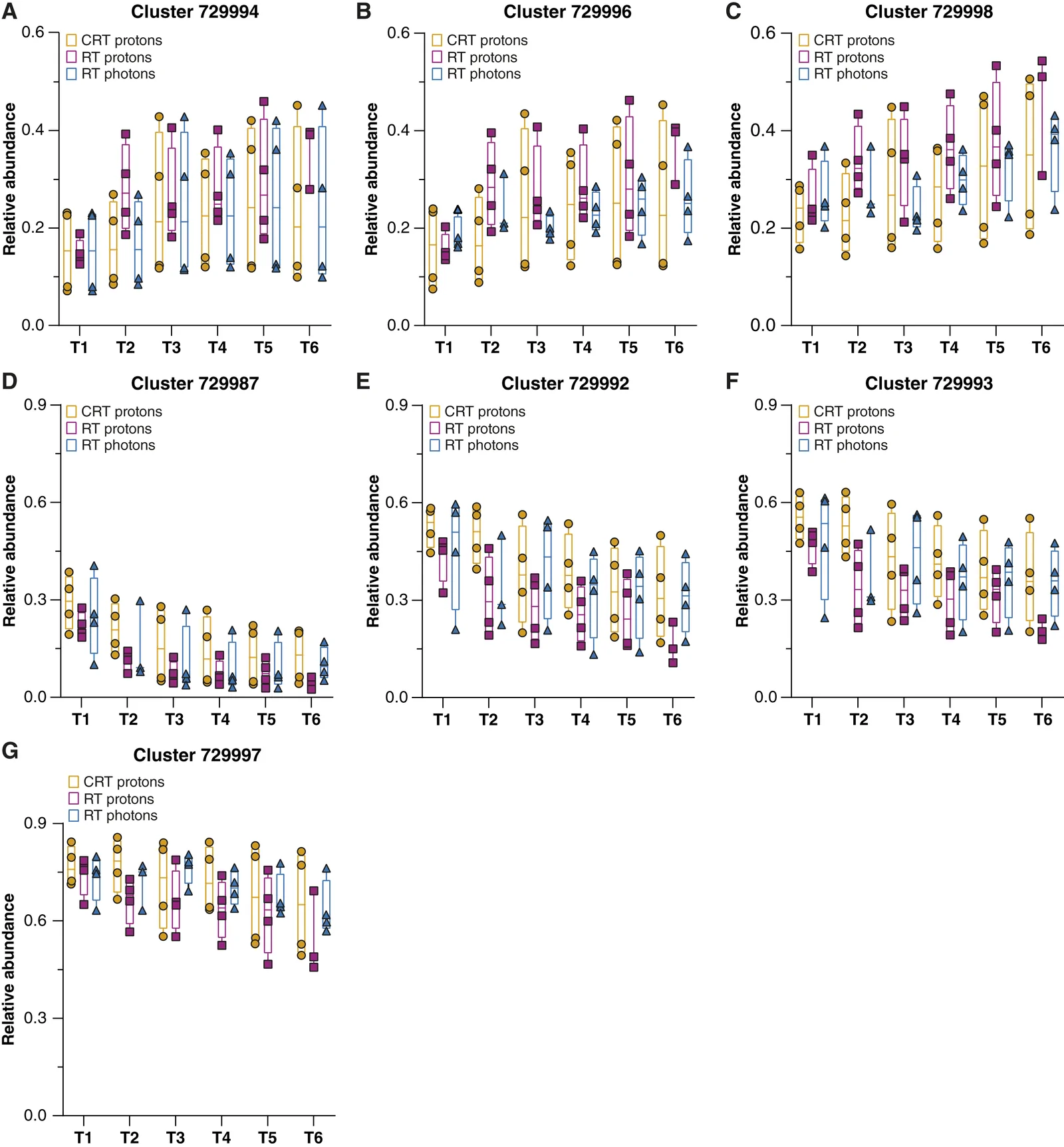
Lymphocyte subsets affected by proton and photon RT. **A–C,** Box plots with whisker plots depicting the increase in relative abundance of NK cells (CD3^−^CD56^+^CD16^+^CD57^+^CD27^−^CD127^−^CD45RA^+^) during and after radiotherapy, represented by clusters (**A**) 729994, (**B**) 729996, and (**C**) 729998. **D–G,** Box plots with whisker plots depicting the significant decrease in the relative abundance of naïve CD4^+^ T cells (CD3^+^CD4^+^CD45RA^+^CCR7^+^CD27^+^CD25^−^CD127^+^CD38^−^CD16^−^CD57^−^) during and after radiotherapy, represented by clusters (**D**) 729987, (**E**) 729992, (**F**) 729993, and (**G**) 729997. T1–6, visit 1–6 [baseline, during treatment, and follow-up visits at 2–26 weeks (i.e., 6 months) after treatment completion, respectively].

Spectral flow cytometry was conducted on circulating lymphocytes from twelve patients, with four representative patients from each treatment group. A statistically significant alteration was observed in seven lymphocyte clusters during (T2) and following radiotherapy (T3–T6; Fig. 4). However, no notable differences were found among the CRT proton, RT proton alone, and RT photon alone treatment groups. Of the seven identified lymphocyte clusters, three clusters showed a significant increase, whereas four demonstrated a significant decrease (Fig. 4A–G). The three lymphocyte clusters showing an increase upon radiotherapy were NK cell populations, characterized by the expression of CD3^−^CD56^+^CD16^+^CD38^+^ (Fig. 4A–C; Supplementary Fig. S2). The four clusters that decreased after radiotherapy belonged to the naïve CD4^+^ T-cell compartment, characterized by the expression of CD3^+^CD4^+^CCR7^+^CD45RA^+^CD27^+^CD127^+^ (Fig. 4D–G; Supplementary Fig. S2). Of interest, this population of naïve CD4^+^ T cells failed to return to baseline levels within 6 months following treatment across all groups.

These data collectively suggest that both photon and proton radiotherapy primarily affect naïve CD4^+^ T cells, with no significant differences between the two RT techniques. Moreover, these effects persist for more than 6 months after treatment completion. The inclusion of cisplatin did not seem to have an additional impact, as similar effects were observed across the treatment groups.

### Effect of proton and photon radiotherapy on memory T-cell responses

**Figure 5.**
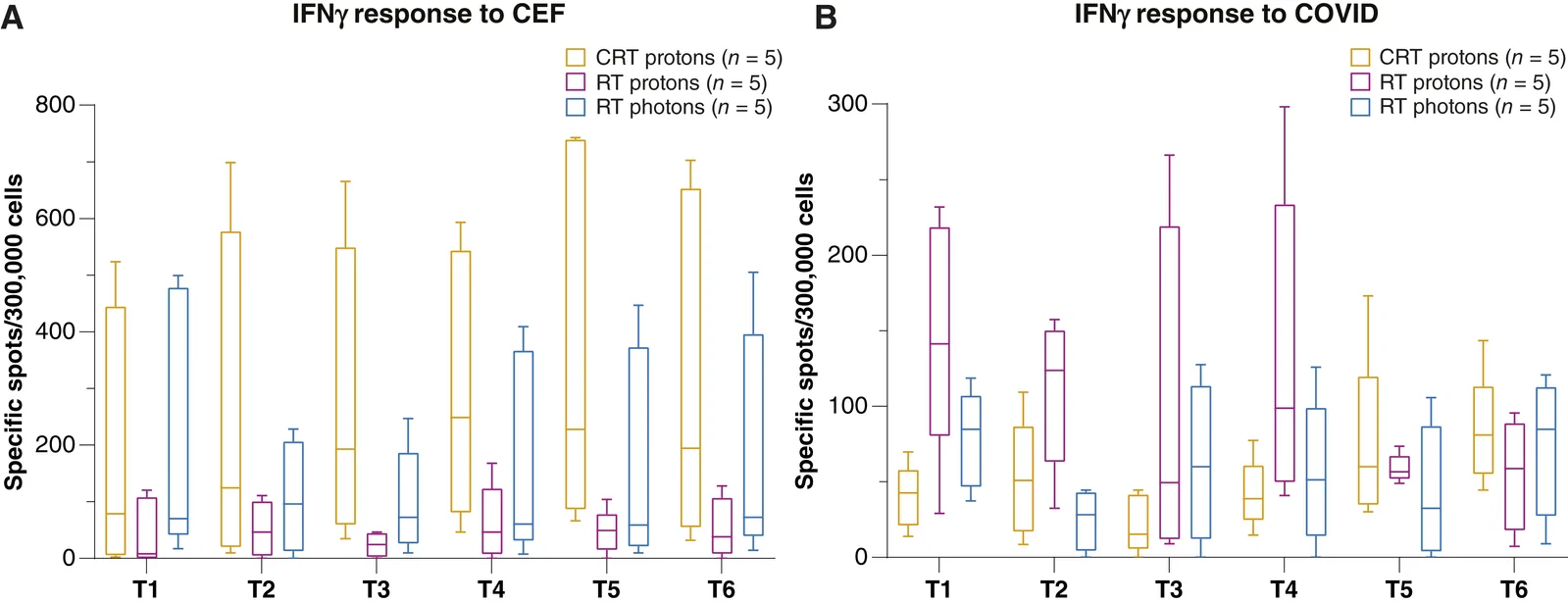
Memory T-cell functionality during and after radiotherapy with protons versus photons. **A** and **B,** Tukey box plots showing the capacity of T cells to respond to (**A**) CEF peptides and (**B**) COVID peptides during and following treatment (up to 6 months after treatment). CEF, 23 different viral peptides of human cytomegalovirus; COVID, 315 peptides derived from the Spike glycoprotein of SARS-CoV-2; EBV, Epstein–Barr virus; flu, influenza A virus; T1–6, visits 1–6 [baseline, during treatment, and follow-up visits at 2–26 weeks (i.e., 6 months) after treatment completion, respectively].

Of the 27 patients, antigen-specific T-cell responses were evaluated in 15 patients, with 5 representative patients selected from each treatment group (Fig. 5; Supplementary Fig. S3).

Three of five patients in the CRT proton group, two of five patients in the RT proton group, and five of five patients in the RT photon group exhibited measurable CEF-specific T-cell responses prior to treatment initiation [T1; Supplementary Fig. S3A–S3C (left)]. Compared with pretreatment levels, no significant differences were observed in CEF-specific T-cell responses within any of the groups during the course of radiotherapy (T2) and the period following the completion of treatment [T3–T6; Fig. 5A; Supplementary Fig. S3A–S3C (left)].

All evaluated patients had measurable COVID-specific T-cell responses prior to treatment initiation [T1; Fig. 5B; Supplementary Fig. S3A–S3C (right)]. Although a trend was observed suggesting a reduction in COVID-specific T-cell responses solely among patients in the RT photon group during the treatment course (T2), no significant alterations in IFNγ responses to COVID were identified within any group throughout both the course of radiotherapy (T2) and the subsequent period following treatment completion (T3–T6), in comparison with pretreatment levels (Fig. 5B).

These data indicate that neither proton nor photon RT affects memory T-cell function. This is in line with the specific depletion of naïve, but not memory T cells, observed using our high-dimensional flow cytometry.

## Discussion

Proton therapy has become a compelling substitute for photon RT in the treatment of HNSCC due to its advantage of delivering lower dosages in off-target regions. However, its impact on circulating immune cells remains poorly understood. In this pilot study, we assessed for the first time the impact of proton RT in comparison with photon RT on circulating immune-cell subsets and their function in patients with stage III to IV HNSCC treated with definitive radiotherapy with protons alone, with photons alone, or with protons in combination with concomitant cisplatin. We demonstrate that across all treatment groups RT, regardless of modality, had substantial immunosuppressive effects on the absolute number of lymphocytes, particularly naïve CD4^+^ T cells, while showing no detectable effects on memory T-cell populations and function.

The present study showed that both proton and photon RT significantly reduced lymphocyte counts in HNSCC. Circulating lymphocytes have been reported to remain markedly depressed for up to 12 months following photon-based radiotherapy in this disease (21–23). Prolonged lymphopenia at 6 to 12 months after photon radiotherapy has been associated with worse recurrence-free survival and OS (22, 23). Therefore, our observation of a more rapid recovery of lymphocyte counts following proton radiotherapy emphasizes its potential advantages for patients with HNSCC.

Studies on esophageal and lung cancers have also compared the impact of proton versus photon RT on circulating lymphocytes, consistently reporting significant decreases in lymphocyte counts with both treatment techniques (14, 15, 24). However, unlike our findings, these studies reported significant differences in lymphocyte counts between the two treatment techniques during the course of treatment, with proton RT demonstrating a more favorable effect on the preservation of lymphocyte counts (14, 15, 24). This discrepancy with our findings may be explained by the high RT doses delivered to the blood pool, including the heart, superior vena cava, and aorta, in esophageal and lung cancers treated with photon RT. Utilizing proton therapy for these tumor types significantly reduces the RT dose to this large blood pool, likely lowering the risk of lymphopenia. This aligns with the proposed mechanism for RT-induced lymphopenia, which is thought to result, among other factors, from the exposure of circulating blood to RT (25). Higher RT doses to the blood pool have been associated with greater lymphocyte depletion (14, 24, 26, 27). The fact that the carotid arteries and tumor-draining lymph nodes (TDLN) are included within the elective treatment volume in HNSCC, for both photon and proton irradiation, might explain the less obvious differences in lymphocyte counts in our study (28).

As expected, patients in our study receiving photon RT were exposed to higher mean doses to the cervical spine vertebral bodies, housing 20% of functional bone marrow (29), and to areas outside the CTV, compared with those treated with proton RT. Notably, patients treated with proton RT demonstrated a faster recovery of lymphocyte counts compared with those receiving photon RT, suggesting that reducing RT exposure of the cervical spine vertebral bodies and tissue outside the CTV may contribute to this effect. Prior studies on proton RT, in which the vertebral bodies were spared, show a smaller decline in lymphocyte counts compared with treatments without vertebral body sparing (17). These findings, together with our observations, suggest that further reduction in RT doses to the vertebral bodies may help mitigate lymphocyte depletion and promote recovery.

To further elucidate the significance of the RT-induced decrease in lymphocytes, high-dimensional and functional immunophenotyping were performed. Currently, no data are available on how proton RT affects lymphocyte subsets and functionality. Our analysis showed that naïve CD4^+^ T cells were the most radiosensitive, whereas NK cells seemed to increase upon radiotherapy among patients with HNSCC subjected to definitive radiotherapy, regardless of the RT technique utilized. This likely reflects their relative radioresistance: As more radiosensitive populations decline, NK cells comprise a larger proportion of circulating PBMCs, even if their absolute numbers remain unchanged. These findings align with prior investigations on photon RT, which identified CD4^+^ T cells, particularly naïve subsets, and B cells as the most radiosensitive populations, whereas NK cells, regulatory T cells, and myeloid cells were relatively radioresistant (21, 30, 31). In our study, both proton and photon RT were observed to negatively affect only naïve CD4^+^ T cells, with no effect on memory T cells. Consequently, it is not surprising that our functional analysis showed no impact of either type of RT on the ability of memory T cells to respond to stimulation with common (recall) viral antigens.

Effective antitumor immunity requires naïve CD4^+^ T cells expressing CD27 (32). These naïve CD4^+^ T cells have the capacity to recognize tumor-associated antigens, facilitate T-cell priming, undergo differentiation into specialized T-helper subsets, and coordinate immune activation. Through these functions, they contribute to the activation of effector CTLs. In fact, in the absence of CD4^+^ T cells, the priming of CD8^+^ T cells is insufficient for their full activation and differentiation into CTLs (33, 34). However, the exact role of circulating CD4^+^ T cells in ICI treatment is not fully established. Although CTLs are the primary mediators of a successful response to ICI, studies in urothelial and renal cell carcinomas have shown that a higher frequency of circulating naïve CD4^+^ T cells is associated with improved therapeutic outcomes (35). Conversely, in lung cancer, the presence of memory CD4^+^ T cells, rather than naïve CD4^+^ T cells, was identified as a strong predictor of treatment response (36). The specific subset of CD4^+^ T cells that is crucial for ICI treatment in HNSCC remains to be determined. Although patients receiving immunotherapy were not included in our study, our observations suggest that RT-induced effects on circulating CD4^+^ T cells may be relevant in the context of ICI treatment.

The combination of radiotherapy and ICI has been explored in HNSCC, predominantly using photon-based techniques, with limited efficacy observed across both recurrent/metastatic and definitive/curative settings (4, 5, 37–40). A key factor constraining therapeutic benefit may be the irradiation of TDLNs, which are essential for antigen presentation and priming of tumor-specific T cells (41). Damage to these structures can impair immune activation and diminish ICI efficacy. Preclinical data suggest that postponing TDLN irradiation until after the critical window of concurrent primary tumor RT and ICI administration may preserve immune function and improve tumor control (42). In line with this concept, the ongoing PRIMO trial (43) is evaluating sentinel lymph node biopsy (SLNB)–guided neck irradiation versus standard bilateral elective irradiation in patients with negative SLNB. If non-inferiority is demonstrated, this approach may support the preservation of lymphocytes and immune structures.

Our study is limited by the small sample size, heterogeneity among patients within and between treatment groups, and its exclusive focus on systemic immunity. Baseline characteristics, such as PS, age, and primary tumor site, were significantly different between treatment groups, with the patients in the CRT proton group being younger and fitter than those in the RT proton and RT photon groups. Age-related immunologic changes may therefore have contributed to the observed differences in immune cell composition and treatment-related dynamics. Aging has been associated with declines in both CD4^+^ and CD8^+^ T-cell populations, although the reduction in CD8^+^ T cells is generally more pronounced than that observed for (naïve) CD4^+^ T cells (44, 45). Notably, in this study, the most prominent treatment-related change was a decline in naïve CD4^+^ T cells following radiotherapy, irrespective of the addition of chemotherapy, including within the relatively younger CRT proton group. This observation suggests that the reduction in naïve CD4^+^ T cells is more likely attributable to radiotherapy-induced lymphocyte depletion than to differences in age distribution between the groups. Although primary tumor sites varied between groups, all patients received bilateral elective neck irradiation as part of standard curative treatment, including nodal levels and adjacent vascular structures. Therefore, although subsite-related factors may have led to some variation in target volume extent, their overall impact on circulating lymphocyte exposure is expected to be limited. To draw more robust conclusions and explore the broader immunologic impact of proton radiotherapy, including potential effects beyond circulating immune cells larger studies are warranted. In particular, future studies should incorporate detailed dosimetric analyses, with a focus on low-dose exposure metrics, as our exploratory findings suggest that parameters such as V2 Gy may be correlated with delayed lymphocyte recovery after radiotherapy.

In conclusion, our findings demonstrate that both proton and photon RT exhibit significant immunosuppressive effects on circulating lymphocytes, mainly on naïve CD4^+^ T cells, during and after (C)RT, lasting for at least 3 months after treatment in locally advanced HNSCC. These results underscore the importance of considering the prolonged immune suppression of radiotherapy after the completion of treatment.

## Supplementary Material

Supplementary Figure S1 Correlations between body and organ-specific radiation exposure and lymphocyte counts.

Supplementary Figure S2 Overview of the characteristics of the seven identified clusters significantly changing upon radiotherapy.

Supplementary Figure S3 Memory T-cell functionality to stimulation with CEF and Covid peptides per patient during and after radiotherapy using protons versus photons.

Supplementary Table S1 Antibodies

Supplementary Table S2 Representativeness of Study Participants

## Data Availability

The data generated in this study are available within the article and its Supplementary Data files. Additional data may contain personally identifying information and are thus not openly available under current General Data Protection Regulation (GDPR) guidelines. Data can be requested from the corresponding author. Data are stored in a controlled access data facility at UMCG, the Netherlands.
