# Supplementary material for: Impact of Proton versus Photon (Chemo)radiation on Circulating Immune Cells in Head and Neck Cancer
Source: Cancer Res Commun. 2026 Sep 8;6(9):2098–109. doi: 10.1158/2767-9764.CRC-26-0496 (PMC13551313; doi:10.1158/2767-9764.CRC-26-0496)
Supplement: Supplementary file 1 [file crc-26-0496_supplementary_figure_s1_suppsf1.pdf]

Supplementary Figure S1

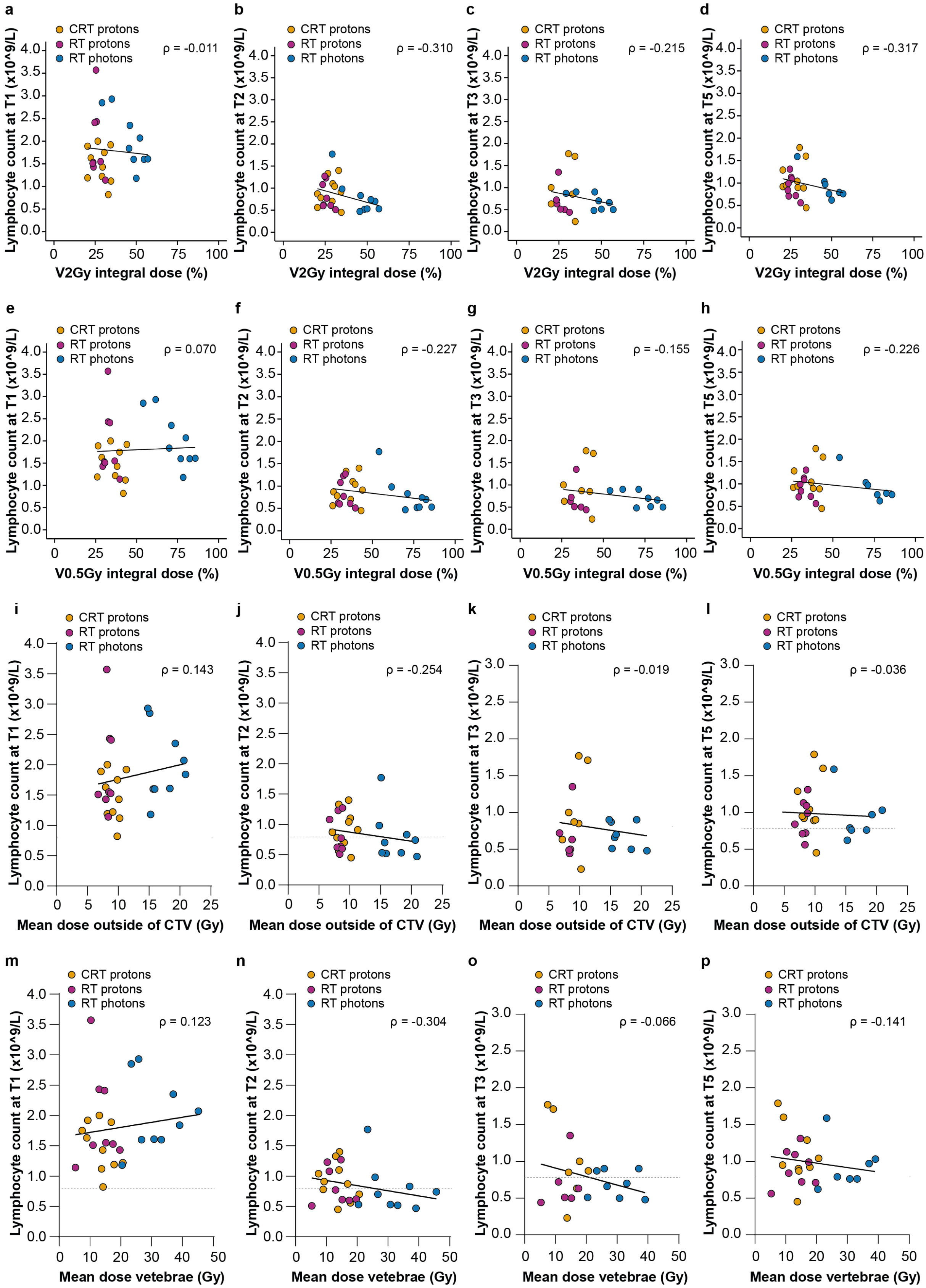

Supplementary Figure S1. Correlations between body and organ-specific radiation exposure and lymphocyte counts. (a-d) Scatterplots showing the correlation between the percentage of body volume receiving 2 Gy of radiation and absolute lymphocyte counts at **(a)** T1, **(b)** T2, **(c)** T3, and **(d)** T5. **(e-h)** Scatterplots showing the correlation between the percentage of body volume receiving 0.5 Gy of radiation and absolute lymphocyte counts at **(e)** T1, **(f)** T2, **(g)** T3, and **(h)** T5. **(i-l)** Scatterplots representing the correlation between radiation dose administered outside of the CTV and the absolute lymphocyte counts at **(i)** T1, **(j)** T2, **(k)** T3, and **(l)** T5. **(m-p)** Scatterplots representing the correlation between radiation dose administered to the vertebral bodies of the cervical spine and the absolute lymphocyte counts at **(m)** T1, **(n)** T2, **(o)** T3, and **(p)** T5. ρ: Spearman correlation coefficient. Dotted line denotes the lower boundary for absolute lymphocyte counts. (C)RT: (chemo)radiation; CTV: clinical target volume; T1: visit 1 (baseline); T2: visit 2 (during treatment); T3 and 5: visit 1 and 5 (follow-up visits at 2 weeks and 3 months after completion of treatment, respectively).
