# Supplementary material for: Impact of Proton versus Photon (Chemo)radiation on Circulating Immune Cells in Head and Neck Cancer
Source: Cancer Res Commun. 2026 Sep 8;6(9):2098–109. doi: 10.1158/2767-9764.CRC-26-0496 (PMC13551313; doi:10.1158/2767-9764.CRC-26-0496)
Supplement: Supplementary file 2 [file crc-26-0496_supplementary_figure_s2_suppsf2.pdf]

Supplementary Figure S2

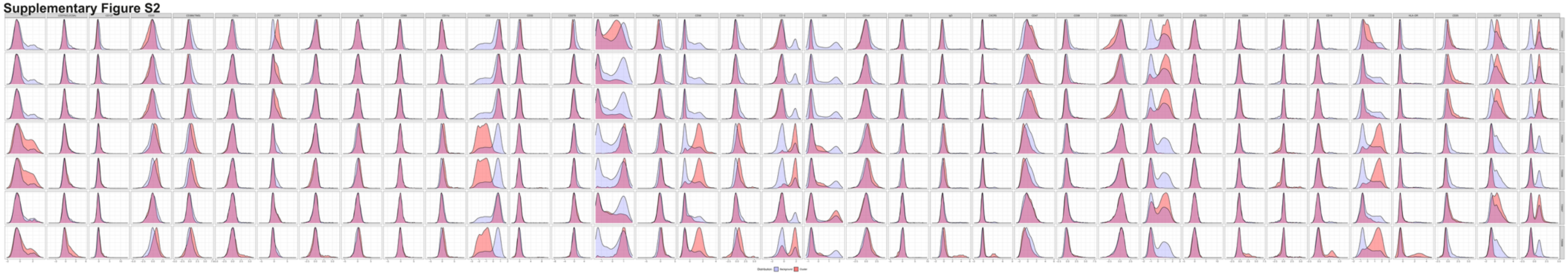

Supplementary Figure S2. Overview of the characteristics of the seven identified clusters significantly changing upon radiotherapy. A population of terminally differentiated cytotoxic NK cells, defined as CD3$^-$CD56$^+$CD16$^+$CD57$^+$CD27$^-$CD127$^-$CD45RA$^+$ and represented by clusters 729994, 729996, and 729998, significantly increased upon treatment with radiotherapy. A population of naïve CD4+ T cells, defined as CD3$^+$CD4$^+$CD45RA$^+$CCR7$^+$CD27$^+$CD25$^-$CD127$^+$CD38$^-$CD16$^-$CD57$^-$ and represented by clusters 729987, 729992, 729993, and 729997, significantly decreased upon radiotherapy. Full size of this figure can be requested at the corresponding author.
