# Supplementary material for: Impact of Proton versus Photon (Chemo)radiation on Circulating Immune Cells in Head and Neck Cancer
Source: Cancer Res Commun. 2026 Sep 8;6(9):2098–109. doi: 10.1158/2767-9764.CRC-26-0496 (PMC13551313; doi:10.1158/2767-9764.CRC-26-0496)
Supplement: Supplementary file 3 [file crc-26-0496_supplementary_figure_s3_suppsf3.pdf]

Supplementary Figure S3

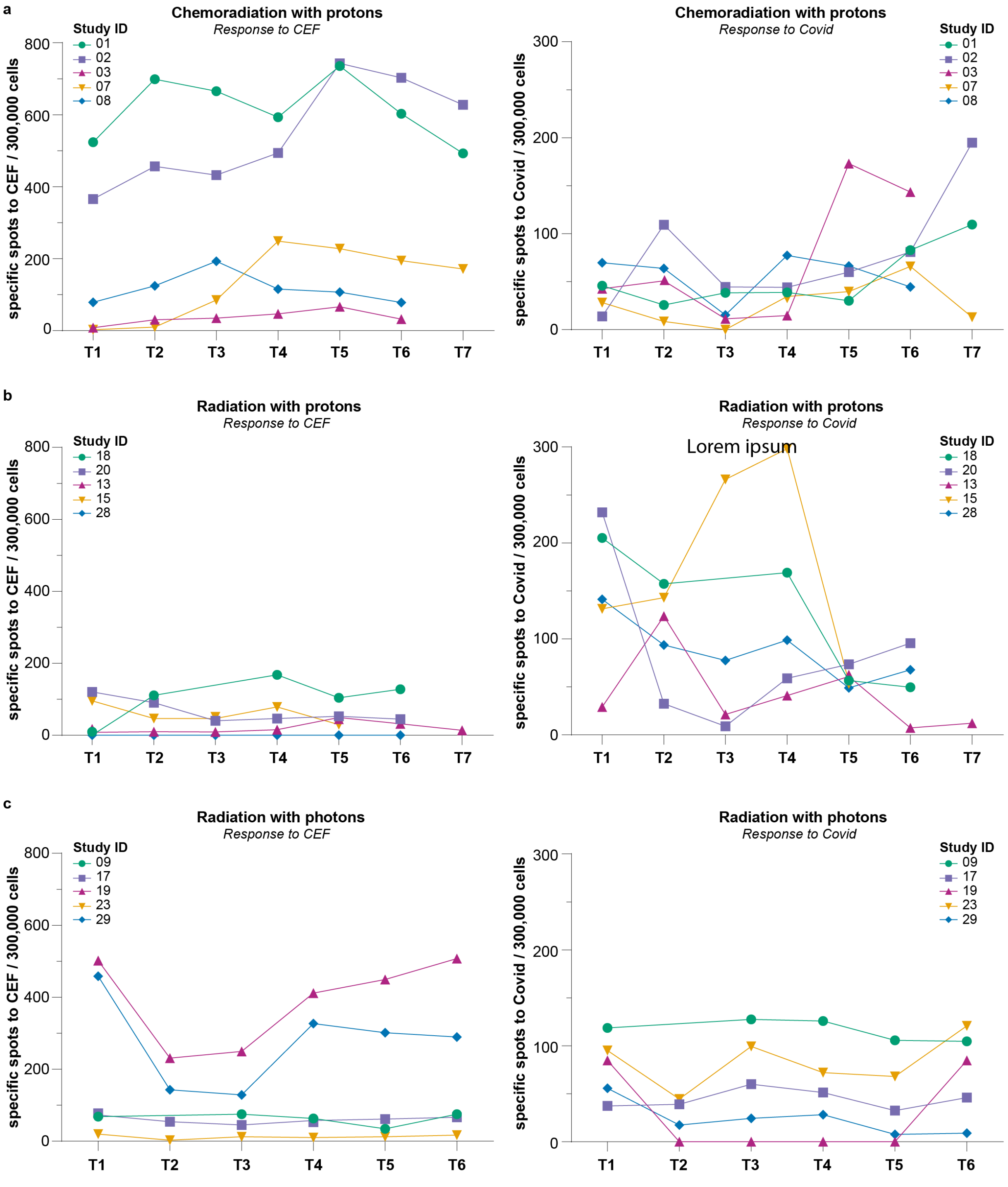

Supplementary Figure S3. Memory T-cell functionality to stimulation with CEF and Covid peptides per patient during and after radiotherapy using protons versus photons. (a-c) IFN-γ T-cell response to stimulation with CEF peptides (left panels) or Covid peptides (right panels) over time ranging from baseline (T1) to 12 months after treatment (T7), graphically depicted for each patient within the **(a)** CRT proton group, **(b)** RT photon group, and **(c)** RT photon group. CEF: 23 different viral peptides of human cytomegalovirus (CMV), Epstein-Barr virus (EBV), and Influenza A virus (flu); Covid: 315 peptides derived from Spike glycoprotein of SARS-CoV-2; (C)RT: (chemo)radiation; T1-7: visit 1-7 (baseline, during treatment, and at follow-up ranging from 2-52 weeks (i.e. 12 months) after treatment completion, respectively).
