# Supplementary material for: Impact of Proton versus Photon (Chemo)radiation on Circulating Immune Cells in Head and Neck Cancer
Source: Cancer Res Commun. 2026 Sep 8;6(9):2098–109. doi: 10.1158/2767-9764.CRC-26-0496 (PMC13551313; doi:10.1158/2767-9764.CRC-26-0496)
Supplement: Supplementary file 4 [file crc-26-0496_supplementary_table_s1_suppst1.pdf]

Supplementary Table S1 | Antibodies					
**Antigen**	**Fluorophore**	**Vendor**	**Catalog**	**RRID**	**Amount per sample**
Live cells	LIVE/DEAD™ Fixable blue	Thermo Fisher	L34962	AB_3717566	1 μl (in 100 μl PBS)
CD123	Super bright	Thermo Fisher	62-1239-42	AB_2662727	2.5 μl
CD11c	efluor 450	Thermo Fisher	48-0116-41	AB_11218498	5 μl
TCR g/d	PerCP-eFluor710	Thermo Fisher	46-9959-41	AB_2573925	5 μl
CD14	spark blue 550	Biolegend	367147	AB_2820021	2.5 μl
CD45	PerCP	Biolegend	368505	AB_2566357	2.5 μl
CD1c	Alexa Fluor 647	Biolegend	331510	AB_1186032	5 μl
CD3	BV 510	Biolegend	344827	AB_2563703	5 μl
CD366 (TIM-3)	PE-Cy5	Biolegend	345051	AB_2819986	5 μl
CD103	PE/Dazzle 594	Biolegend	350223	AB_2716188	5 μl
CD279 (PD-1)	BV 785	Biolegend	329929	AB_11218984	5 μl
CD57	FITC	Biolegend	359603	AB_2562386	1.25 μl
CD197 (CCR7)	BV 421	Biolegend	353207	AB_10915137	10 μl
CD370	PE	Biolegend	353803	AB_10959651	5 μl
CD25	PE/Fire 700	Biolegend	356145	AB_2876678	5 μl
CD38	APC/Fire 810	Biolegend	356643	AB_2860936	5 μl
IgM	BV 570	Biolegend	314517	AB_10913816	3 μl
CD11b	PerCP-Cy5.5	Biolegend	301327	AB_10900072	5 μl
CD27	APC/Fire 750	Biolegend	302845	AB_2632664	10 μl
CD19	Spark NIR 685	Biolegend	302269	AB_2860769	5 μl
CXCR5 (CD185)	BV750	BD	747111	AB_2871862	1.25 μl
CD303	BUV563	BD	748415	AB_2872834	2.5 μl
CD47	BUV615	BD	751336	AB_2875344	2.5 μl
CD45RA	BUV395	BD	740315	AB_2740052	1.25 μl
CD39	BUV661	BD	749967	AB_2874196	5 μl
CD4	NovaFluor Yellow 570	Thermo Fisher	H001T02Y01	AB_2896369	2 μl
HLA-DR	PE/Fire 810	Biolegend	900000155	AB_2904336	5 μl
CD127	R718	BD	566967	AB_2869977	10 μl
CD141	BB515	BD	566017	AB_2739462	5 μl
CD8	BUV805	BD	612889	AB_2833078	5 μl
CD16	BUV496	BD	612944	AB_2870224	5 μl
CD33	BV711	BD	563171	AB_2738045	5 μl
IgD	BV480	BD	566187	AB_2739583	5 μl
IgG	BV605	BD	563246	AB_2738092	5 μl
CD137	PE-Cy7	Biolegend	309817	AB_2287731	5 μl

CD56	BUV737	BD	612766	AB_2813880	5 µl
CD69	BV650	BD	563835	AB_2738442	5 µl
CD24	PE-Alexa Fluor 610	Thermo Fisher	MHCD2422	AB_10375871	5 µl
CD34	APC	Biolegend	343607	AB_207435	5 µl
CD20	pacific orange	Thermo Fisher	MHCD2030	AB_10375578	5 µl
