# Supplementary material for: Impact of Proton versus Photon (Chemo)radiation on Circulating Immune Cells in Head and Neck Cancer
Source: Cancer Res Commun. 2026 Sep 8;6(9):2098–109. doi: 10.1158/2767-9764.CRC-26-0496 (PMC13551313; doi:10.1158/2767-9764.CRC-26-0496)
Supplement: Supplementary file 5 [file crc-26-0496_supplementary_table_s2_suppst2.pdf]

Supplementary Table S2 | Representativeness of Study Participants	
**Disease**	Head and neck squamous cell carcinoma (HNSCC)
**Consideration related to:**	
Sex	HNSCC affects both men and women but is approximately twice as common in men.
Age	The median age at the time of diagnoses is 64, with more than half of the patients diagnosed at an age older than 60 years.
Race/ethnicity	HNSCC affects individuals across all racial and ethnic groups. In Western countries approximately 70-80% of cases occur in Caucasians. Patients with a lower socioeconomic status and poor oral hygiene are at higher risk.
Geography	HNSCC is observed across all areas of the world. Our study was conducted in the Netherlands only, where approximately 3,200 new cases are diagnosed each year, with around 1,000 deaths annually.
Other consideration	About 20% of all HNSCC cases are HPV-positive, rising to 40-60% in oropharyngeal cancers. These tumors generally exhibit a more inflamed immune microenvironment than HPV-negative HNSCCs.
Overall representativeness of this study	The age distribution of HNSCC of our study aligns with that reported in literature (55-74 years) and in the Netherlands (60-74 years). Similarly, the sex distribution mirrors previous findings, with a male-to-female ratio of approximately 2:1.<br>However, because our study was conducted at a single center in the northern region of the Netherlands, all participants were Caucasians. While HNSCC is more prevalent among Caucasians, this homogeneity may limit the study's overall representativeness.
